# Microcirculation and blood transfusion: effects of three different types of concentrated red blood cells - preliminary results

**DOI:** 10.1186/cc10811

**Published:** 2012-03-20

**Authors:** A Donati, E Damiani, R Domizi, C Scorcella, A Carsetti, MR Lombrano, V Fiori, P Pelaia

**Affiliations:** 1 Università Politecnica delle Marche, Ancona, Italy

## Introduction

Red blood cell (RBC) transfusions are used to increase oxygen delivery; however, a restrictive transfusion strategy (predefined hemoglobin threshold of 7 g/dl) was demonstrated to be associated with lower mortality and incidence of nosocomial infections than a liberal one [1,2]. This may be related to the storage process, which could affect the ability of RBCs to transport and delivery oxygen, or to immunomodulating effects of cytokines from residual leukocytes [2]. The aim of the study is to evaluate the effects, on microcirculation of septic patient, of three types of RBCs.

## Methods

A controlled randomized prospective study on 45 patients with sepsis, severe sepsis or septic shock requiring RBC transfusion. Patients are randomized into three groups receiving: (1) fresh standard RBCs (storage <10 days); (2) leukodepleted RBCs; and (3) old standard RBCs (storage >20 days) respectively. Before and 1 hour after the transfusion, microcirculation is evaluated using sidestream dark-field imaging [3] and near-infrared spectroscopy with a vascular occlusion test. We also monitor temperature, heart rate, mean blood pressure, hemochrome, blood gases, blood lactates and SOFA score.

## Results

**Figure 1:**
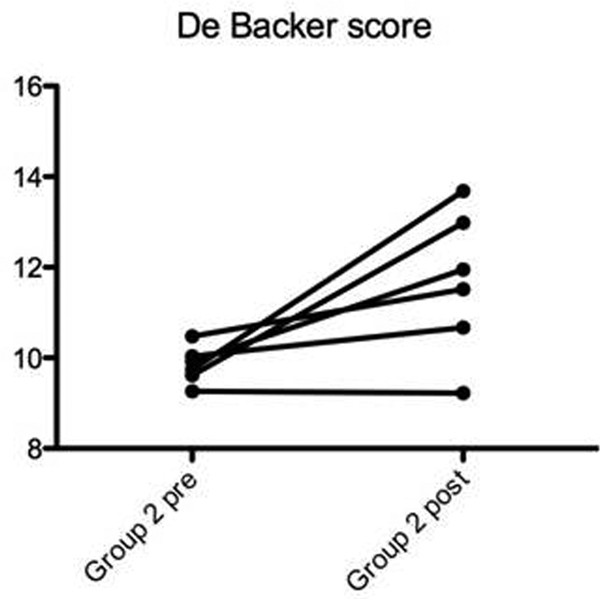
**De Backer score pre and post transfusion in group 2**.

Preliminary data on 18 patients, six for each group: before and after transfusion, in group 2, but not in groups 1 and 3, there is a trend to an increase in MFIs (*P *= 0.09), DeBacker score (Figure 1, *P *< 0.05), PPV (*P *= 0.07) and PVD (*P *= 0.07). No relevant differences for other parameters.

## Conclusion

After transfusion, microcirculation seems to be improved in the leukodepleted RBC group with a significant improvement of De Backer score and a trend to improve the other microcirculatory parameters, while in the other three groups there was not this trend.

## References

[B1] Hebert PC N Engl J Med 1999 340 409 417 10.1056/NEJM199902113400601 9971864

[B2] Rosemary L Blood Transfusion 2010 8 Suppl 3 S2620606746

[B3] De Backer D Crit Care 2007 11 R101 10.1186/cc6118 17845716 PMC2556744

